# Identification of Key Genes and Pathways Associated with Sex Differences in Osteoarthritis Based on Bioinformatics Analysis

**DOI:** 10.1155/2019/3482751

**Published:** 2019-12-06

**Authors:** Shiying Wang, Huanmei Wang, Wei Liu, Biaofang Wei

**Affiliations:** ^1^Guangzhou University of Chinese Medicine, Guangzhou, Guangdong Province 510006, China; ^2^Department of Femoral Head, Linyi People's Hospital, Linyi, Shandong Province 276000, China; ^3^Department of Rehabilitation, The Ninth Hospital of Wuhan, Wuhan, Hubei Province 430000, China; ^4^Department of Encephalopathy, The Second Affiliated Hospital of Shandong University of Traditional Chinese Medicine, Jinan, Shandong 250001, China

## Abstract

Sex differences have been suggested to play critical roles in the pathophysiology of osteoarthritis (OA), resulting in sex-specific prevalence and incidence. However, their roles in the development of OA remain largely unknown. The aim of this study was to screen out key genes and pathways mediating biological differences between OA females after menopause and OA males. First, the gene expression data of GSE36700 and GSE55457 were downloaded from the Gene Expression Omnibus database. Differentially expressed genes (DEGs) between sexes were identified using R software, respectively. The overlapping DEGs were obtained. Then, protein-protein interactive (PPI) network was constructed to further analyze interactions between the overlapping DEGs. Finally, enrichment analyses were separately performed using Gene Ontology and Kyoto Encyclopedia of Genes and Genomes tools. In our results, a total of 278 overlapping DEGs were identified between OA females after menopause and OA males, including 219 upregulated and 59 downregulated genes. In the PPI network, seven hub genes were identified, including EGF, ERBB2, CDC42, PIK3R2, LCK, CBL, and STAT1. Functional enrichment analysis revealed that these genes were mainly enriched in PI3K-Akt signaling pathway, osteoclast differentiation, and focal adhesion. In conclusion, the results in the current study suggest that pathways of PI3K-Akt, osteoclast differentiation, and focal adhesion may play important roles in the development of OA females after menopause. EGFR, ERBB2, CDC42, and STAT1 may be key genes related to OA progression in postmenopausal women and may be promising therapeutic targets for OA.

## 1. Introduction

Osteoarthritis (OA), the most common musculoskeletal disorder, leads to functional disability and loss in quality of life. It affects women after menopause 2-3 times often than men [1]; worldwide disease estimates show that approximately 18% of women and 6% of men at the age of 60 years or older suffer from OA [2]. It is characterized by cartilage degradation, synovial inflammation, subchondral bone sclerosis, and chronic pain [3, 4]. Unfortunately, adequate therapies cannot block or reverse OA progression apart from pain relief [5]. Eventually, joint replacement surgery is commonly applied to patients with advanced stage. It is therefore urgent to explore potential biomarkers and therapeutic targets for OA.

Sex differences in its prevalence suggest a significant role for sex hormones in the pathogenesis of OA. This has led to the hypothesis that female sex hormones may have protective effects on the risk of developing OA. A number of studies have assessed the association between sex hormone levels and the risk of OA, but with conflicting results. One observational study showed that decreased sex hormone levels were associated with an increased prevalence of hand OA in nonelderly females [6]. Spector et al. [7] reported that middle-aged women with OA had lower circulating sex hormone levels compared with healthy women. By contrast, a cohort study showed greater log-transformed concentrations of sex hormones were associated with a higher incidence of hip replacement for OA [8]. Overall, previous evidence for the sex-specific mechanism in OA is inconclusive.

Recently, microarray technology based on high-throughput platforms has been widely used to profile gene expression. A lot of effort has been spent in identifying or assessing specific genes and pathways for the progression of OA. The majority of studies are focused on gene expression data in OA patients compared to healthy subjects [9–13]. But there is less information regarding the differences of gene expression data between sexes. Thus, the purpose of this study was to identify key genes and pathways contributing to biological differences between sexes by a comprehensive bioinformatics analysis. This study may improve the understanding of the sex-specific mechanism in OA and suggest potential biomarkers and therapeutic targets for OA treatment.

## 2. Materials and Methods

### 2.1. Microarray Data

In this study, we searched datasets from the Gene Expression Omnibus (GEO) repository (https://www.ncbi.nlm.nih.gov/geo/) with the keywords: “osteoarthritis” [MeSH Terms] OR “osteoarthritis” [All Fields] AND “Homo sapiens” [porgn] AND “gse”[Filter]. The screening standards were as follows: the microarray datasets were gene expression profiles; the clinical information in datasets contains gender and age. Eventually, two datasets were screened out for further analysis: GSE36700 [14] and GSE55457 [15]. Both datasets include synovial membrane samples from OA females and OA males. GSE36700 is based on GPL570 [HG-U133_Plus_2] Affymetrix Human Genome U133 Plus 2.0 Array (Affymetrix UK Ltd., High Wycombe, UK). GSE55457 is based on GPL96 [HG-U133A] Affymetrix Human Genome U133A Array (Affymetrix, Santa Clara, California, USA). According to our aims, only OA females after menopause and OA males in both datasets were selected for further analysis in the present study.

### 2.2. Differential Expression Analysis

We used software R (version 3.5.1) and related packages to compare differentially expressed genes (DEGs) between OA females after menopause and OA males in GSE36700 and GSE55457, respectively. Both datasets were first background adjusted and normalized by log_2_ transformation. Background correction, quantile normalization, and probe summarization of the raw microarray data were performed by robust multiarray average (RMA) algorithm [16] in Linear Models for Microarray Data (limma) package [17]. The detailed analysis was according to the manufacturer's protocol. Then, *P* values were adjusted for comparisons using the false discovery rate (FDR) of the Benjamini and Hochberg (BH) test [18] in limma package [17]. DEGs were selected with the commonly used thresholds of a *P* value <0.05 and |log_2_fold change(FC)| > .5 [19, 20]. Volcano plot of DEGs was generated by ggplot2 package in R software. Furthermore, the overlapping DEGs were the intersection of DEGs in two datasets.

### 2.3. Functional Enrichment Analysis

The Database for Annotation, Visualization, and Integrated Discovery (DAVID, version 6.8, https://david.ncifcrf.gov/) [21] is a well-known online tool that provides comprehensive information for list of DEGs by Gene Ontology (GO) and the Kyoto Encyclopedia of Genes and Genomes (KEGG) pathway analyses. In order to obtain the biological function and signaling pathways of DEGs, we undertook GO enrichment and KEGG enrichment analyses for DEGs through DAVID. Only enriched gene count of GO and KEGG terms more than or equal to 3 and a *P* value <0.05 were considered as statistically significant.

### 2.4. Protein-Protein Interaction (PPI) Network Analysis

In order to identify functional interactions between the products of the overlapping DEGs, the overlapping DEGs were uploaded to the Search Tool for the Retrieval of Interacting Genes (STRING, version 11.0, https://www.string-db.org/) [22], which is an open accessible online database of genetic and protein interactions. The PPI network was constructed via STRING with the default threshold of a combined score >0.4. Then, we performed Cytoscape software (version 3.7.2) [23] to visualize the PPI network. In addition, nodes in the PPI network represent proteins, while the edges represent the interactions. The hub genes were those highly connected proteins possessing important biological function with a node degree greater than or equal to 10.

## 3. Results

### 3.1. Identification of Overlapping DEGs

**Figure 1:**
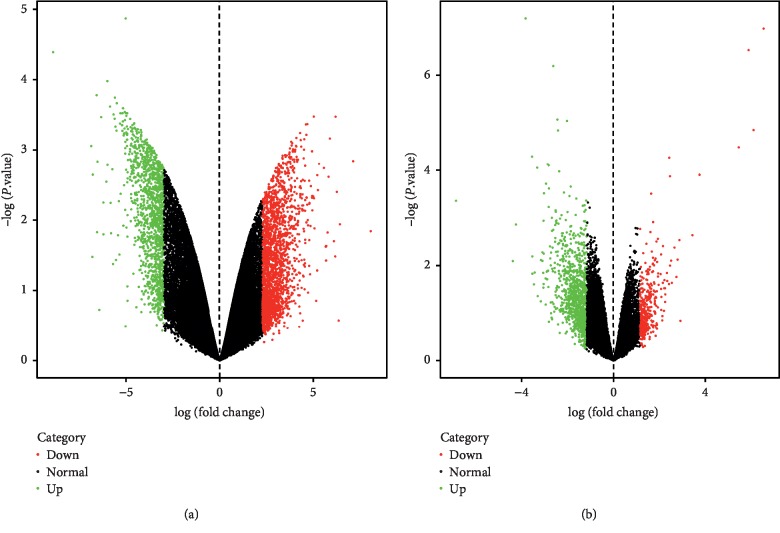
Volcano plots of DEGs in OA females after menopause versus OA males in (a) GSE36700 and (b) GSE55457.

**Figure 2:**
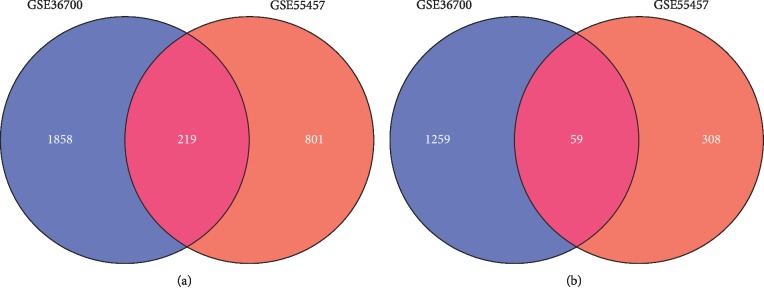
Venn diagrams of (a) upregulated and (b) downregulated overlapping DEGs in GSE36700 and GSE55457.

A total of 3395 and 1387 DEGs were identified in synovial membranes from OA females after menopause compared with OA males in GSE36700 and GSE55457, respectively. Among them, 2077 upregulated and 1318 downregulated genes were obtained from GSE36700; meanwhile, 1020 upregulated and 367 downregulated genes were obtained from GSE55457. The volcano plots are shown in Figure 1, in which the red represents upregulated genes and the green represents downregulated genes. Furthermore, a total of 278 overlapping genes were identified in two datasets after the intersection, including 219 upregulated and 59 downregulated genes. As shown in Figure 2, the Venn diagrams displayed the overlapping genes in two datasets after the intersection.

### 3.2. GO Enrichment Analysis

**Figure 3:**
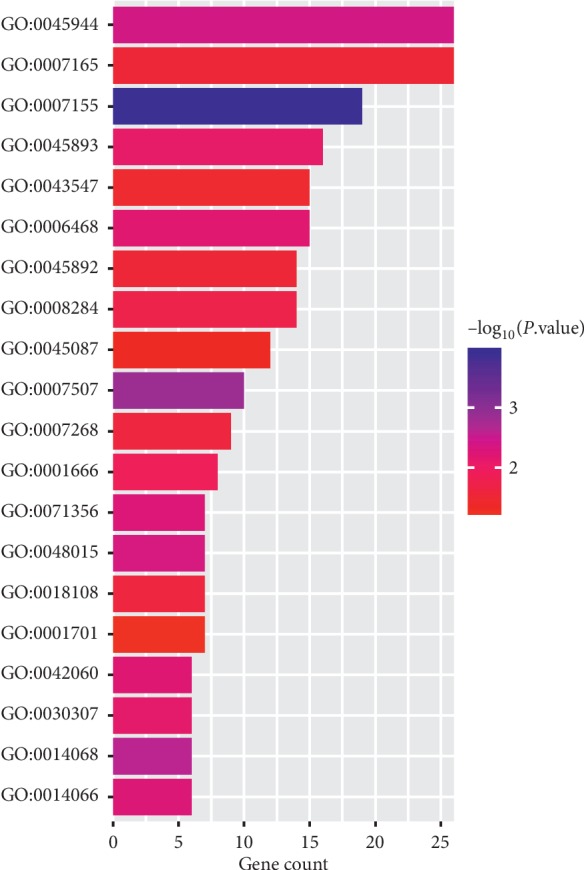
The top 20 GO enrichment analysis of the overlapping DEGs of OA females after menopause versus OA males.

The GO enrichment analysis was performed to determine the biological function of the overlapping DEGs. A total of 50 GO terms are remarkably enriched for the overlapping DEGs. The overlapping DEGs were mainly enriched in positive regulation of transcription from RNA polymerase II promoter, signal transduction, cell adhesion, positive regulation of transcription, DNA-templated, protein phosphorylation, positive regulation of GTPase activity, positive regulation of cell proliferation, and regulation of phosphatidylinositol 3-kinase signaling. The top 20 GO terms are shown in Figure 3 and Table 1.

### 3.3. KEGG Pathways Analysis

**Figure 4:**
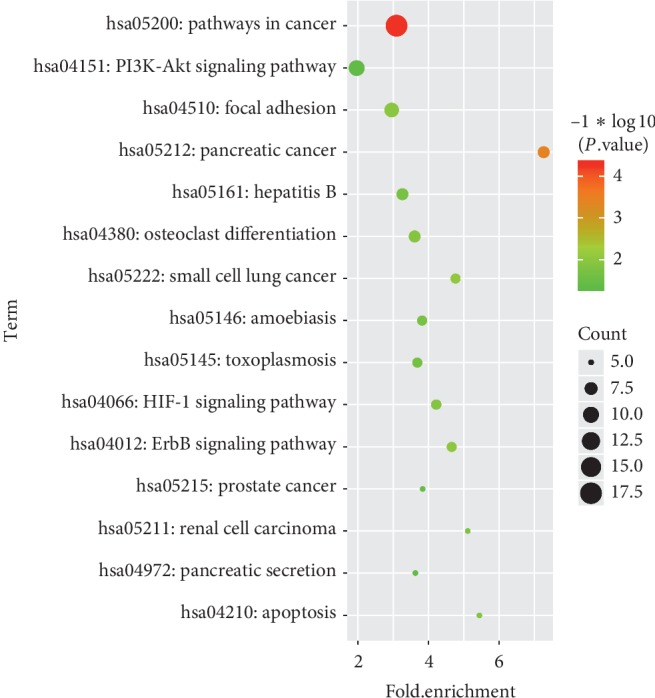
Enriched pathways of the overlapping DEGs in OA females after menopause and OA men.

KEGG pathways analysis was performed to explore the enriched pathways of the overlapping DEGs. As shown in Figure 4, a total of 15 pathways are identified. The pathways related to sex differences in OA are mainly enriched as follows: PI3K-Akt signaling pathway (*P* value = 4.59*E* − 02, which involved epidermal growth factor (EGF) and phosphoinositide-3-kinase regulatory subunit 2 (PIK3R2)), osteoclast differentiation (*P* value = 1.23*E* − 02, which involved PIK3R2 and signal transducer and activator of transcription 1 (STAT1)), and focal adhesion (*P* value = 3.10*E* − 02, which involved EGF, PIK3R2, and erb-b2 receptor tyrosine kinase 2 (ERBB2)).

### 3.4. PPI Network Construction

**Figure 5:**
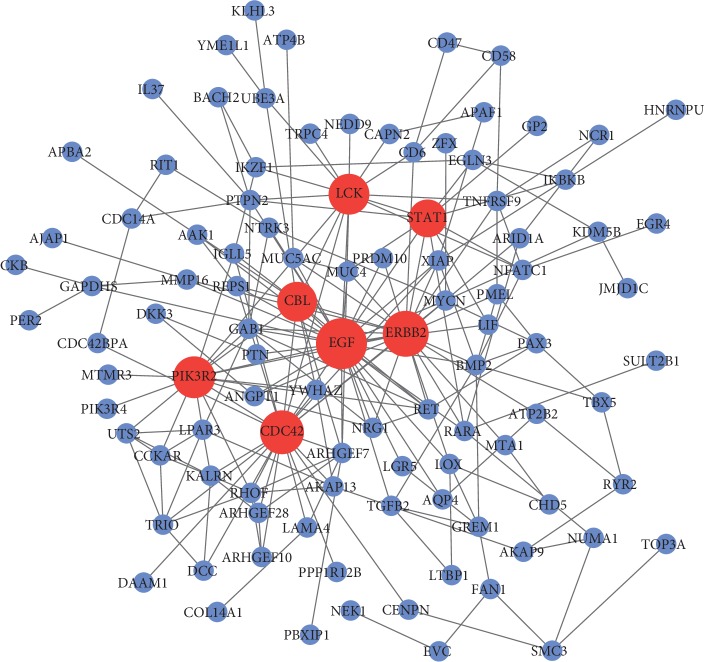
PPI network of the overlapping DEGs in OA females after menopause and OA men.

To aid in the understanding of the interactions between the overlapping DEGs, PPI network was constructed using the STRING database. The PPI network was composed of 243 nodes (the overlapping DEGs) and 330 edges (interactions between the overlapping DEGs). The genes with higher scores were the hub genes, as the genes of higher degree may be associated with OA. As displayed in Figure 5, the hub genes with node degree greater than or equal to 10 are EGF (degree = 30), ERBB2 (degree = 25), cell division cycle 42 (CDC42) (degree = 24), PIK3R2 (degree = 17), LCK protooncogene, Src family tyrosine kinase (LCK) (degree = 15), Cbl protooncogene B (CBL) (degree = 12), and STAT1 (degree = 11). Among them, EGF, ERBB2, PI3KR2, and LCK were upregulated in OA postmenopausal women compared to OA men; meanwhile, CDC42, CBL, and STAT1 were downregulated in OA postmenopausal women compared to OA men.

## 4. Discussion

OA is a degenerative joint disease with sex-specific prevalence and incidence. The prevalence of OA is higher among women than among men, and the risk for developing OA increases among women after menopause [24]. Sex hormones help in explaining sex differences in OA, albeit partly. After menopause, the level of estrogen is reduced continuously while the incidence of OA is rising dramatically, suggesting estrogen may have a protective effect on OA. Unfortunately, long-term use of estrogen cannot block or reverse OA progression but increases the risk of cancers such as breast cancer and endometrial cancer [25, 26]. The underlying mechanisms contributing to sex differences in OA are still poorly understood. It is important to study the molecular mechanisms of sex differences in OA.

To our knowledge, our study, for the first time, identified key genes and pathways in synovial membrane of OA females after menopause compared to OA males using bioinformatics analysis. In the present study, we performed an integrated bioinformatics analysis using two GEO datasets of gene expression profiles to identify the biological mechanisms involved in the pathogenesis of sexes differences in OA. A total of 278 overlapping DEGs were identified between OA postmenopausal women and OA men, including 219 upregulated and 59 downregulated ones. Among the overlapping DEGs, EGF, ERBB2, CDC42, PIK3R2, LCK, CBL, and STAT1 were identified as the hub genes. GO enrichment analysis of the overlapping DEGs showed that the hub genes EGF, ERBB2, and PIK3R2 were mainly enriched in regulation of phosphatidylinositol 3-kinase (PI3K) signaling, positive regulation of GTPase activity, and phosphatidylinositol-mediated signaling. Besides, KEGG pathways of the overlapping DEGs revealed that these hub genes were mainly enriched in pathways of PI3K-Akt, focal adhesion, and osteoclast differentiation. The results of the present study were reasonable and consistent with the results of previous studies, which have also suggested that PI3K-Akt signaling pathway [27–29], focal adhesion [30], and osteoclast differentiation [31, 32] are involved in the pathogenesis of OA.

In the PPI network construction, we screened out seven hub genes EGF, ERBB2, CDC42, PIK3R2, LCK, CBL, and STAT1. All these genes may play important roles in the occurrence and progression in OA, particularly in OA females after menopause. Mounting evidence highlights an important role of EGF in the pathogenesis of OA. EGF is an active polypeptide composed of 53 amino acids. Epidermal growth factor receptor (EGFR, ERBB1) is a member of the receptor tyrosine kinases (RTKs) family which also includes ERBB2/HER2, ERBB3/HER3, and ERBB4/HER4. EGFR, with intrinsic tyrosine kinase activity, can be bound and activated by a family of several peptide growth factors including EGF [33]. Compared with EGFR, ERBB2 does not contain a ligand-binding domain. The activation of EGFR occurs through trans-autophosphorylation of the intracellular kinase domain. Phosphorylated EGFR initiates myriad of key signaling pathways, among which the most studied are PI3K/Akt, JAK/STAT, and so on. In addition, PI3K/Akt acts as one of the vital signaling pathways in the development of OA [34]. In non-small-cell lung cancer, high expression of EGFR correlates with poor survival and anti-EGFR agents have greatly improved the progression-free survival over standard chemotherapy [35]. In breast cancer, overexpression of HER2 is generally associated with poor prognosis and HER2 targeted therapies have significantly improved the therapeutic outcome [36]. In the late stage of OA, human OA samples contain significantly more activated EGFR than controls; gefitinib could efficiently inhibit EGFR functions in OA joints and restore cartilage structure and function in the mouse model [37]. As to ERBB2, it is highly expressed in rheumatoid arthritis synovial cells compared to controls [38]. Similarly, CDC42, a member of Rho family small GTPases, is highly expressed in both articular cartilage and subchondral bone in a mouse OA model; knockdown of Cdc42 expression or inhibition of Cdc42 activity robustly attenuates knee joint destruction in mouse [39]. STAT1 has been widely regarded as a significant transcription factor involved in joint inflammation and destruction [40]. In addition to the genes that have already been discussed, there is a lack of data regarding whether PI3KR2, LCK, and CBL have strong connections with OA occurrence and progression. Given the specific pathophysiologic roles of EGFR, ERBB2, CDC42, and STAT1 in the development of joints, and investigation into targeted inhibition of these receptors in the setting of OA is of great interest.

The pathogenesis of OA is multifactorial and intricate and is characterized by cartilage destruction, synovial inflammation, and subchondral bone sclerosis. It is widely accepted that the PI3K/Akt signaling pathway plays a critical role in regulating diverse cellular processes including cell proliferation, apoptosis, metabolism, differentiation, and cell cycle [41]. Activation of PI3K signal transduction pathway has been recently recognized to be one of the important mechanisms for antiapoptosis of cells. Researchers found that activation of PI3K-Akt signaling pathway can increase matrix metalloproteinase (MMP) production which results in the progression of OA, while inhibition of PI3K-Akt signaling pathway attenuates the development of OA [34]. Further researches indicated that inhibition of PI3K-Akt signaling pathway attenuates inflammatory response in rats with OA [42]. In the current study, the upregulated gene PI3KR2 was enriched in PI3K-Akt signaling pathway in OA postmenopausal women. Prompted by the overexpressed PI3KR2 in cancers which correlates with PI3K activation [43], we could assume that the upregulated PI3KR2 might be related to the activation of PI3K-Akt signaling pathway in OA. Subsequently, the activated PI3K-Akt pathway contributes to the occurrence and development of OA. This may partly explain why OA occurs more frequently in postmenopausal women than in men. Yet, this is still required to be further confirmed by additional biochemistry and clinic-related studies.

The KEGG pathways of the overlapping DEGs revealed that the hub genes were mainly enriched in osteoclast differentiation and focal adhesion as well. It is now well established that in OA, the balance between osteoclast-induced bone resorption and osteoblast-induced remodeling is being progressively deregulated [44]. Osteoclasts are multinucleated cells derived from hematopoietic stem cells of monocytic line and specialized for bone resorption. Studies have found increased bone resorption in patients with progressive OA compared to nonprogressive OA [45]. Our findings for these enrichments in OA suggest that osteoclast differentiation and focal adhesion may play an important part in the development of OA, especially in postmenopausal patients.

In summary, the results in the current study suggest that pathways of PI3K-Akt, osteoclast differentiation, and focal adhesion may play important roles in the development of OA females after menopause. EGFR, ERBB2, CDC42, and STAT1 may be key genes related to OA progression in postmenopausal women and may be promising therapeutic targets for OA.

## Figures and Tables

**Table 1:** The top 20 GO enrichment analysis of the overlapping DEGs associated with sex in OA.

GO term	Biological function	Gene count	*P* value
GO:0045944	Positive regulation of transcription from RNA polymerase II promoter	26	3.86*E* − 03
GO:0007165	Signal transduction	26	2.76*E* − 02
GO:0007155	Cell adhesion	19	1.18*E* − 04
GO:0045893	Positive regulation of transcription, DNA-templated	16	7.62*E* − 03
GO:0006468	Protein phosphorylation	15	6.31*E* − 03
GO:0043547	Positive regulation of GTPase activity	15	3.39*E* − 02
GO:0008284	Positive regulation of cell proliferation	14	1.75*E* − 02
GO:0045892	Negative regulation of transcription, DNA-templated	14	2.80*E* − 02
GO:0045087	Innate immune response	12	4.65*E* − 02
GO:0007507	Heart development	10	1.33*E* − 03
GO:0007268	Chemical synaptic transmission	9	2.28*E* − 02
GO:0001666	Response to hypoxia	8	1.22*E* − 02
GO:0048015	Phosphatidylinositol-mediated signaling	7	4.25*E* − 03
GO:0071356	Cellular response to tumor necrosis factor	7	5.09*E* − 03
GO:0018108	Peptidyl-tyrosine phosphorylation	7	2.32*E* − 02
GO:0001701	In utero embryonic development	7	5.29*E* − 02
GO:0014068	Positive regulation of phosphatidylinositol 3-kinase signaling	6	2.40*E* − 03
GO:0014066	Regulation of phosphatidylinositol 3-kinase signaling	6	5.27*E* − 03
GO:0042060	Wound healing	6	5.86*E* − 03
GO:0030307	Positive regulation of cell growth	6	7.19*E* − 03

## Data Availability

The data used to support the findings of this study are available from the corresponding author upon request.
